# Two-stage joint selection method to identify candidate markers from genome-wide association studies

**DOI:** 10.1186/1753-6561-3-s7-s29

**Published:** 2009-12-15

**Authors:** Zheyang Wu, Chatchawit Aporntewan, David H Ballard, Ji Young Lee, Joon Sang Lee, Hongyu Zhao

**Affiliations:** 1 Department of Epidemiology and Public Health, Yale University, 60 College Street, New Haven, Connecticut 06051, USA; 2 Department of Psychiatry, Yale University, 300 George Street, New Haven, Connecticut 06511, USA; 3 Program in Computational Biology and Bioinformatics, Yale University, P.O. Box 208114, New Haven, Connecticut 06520-8114, USA; 4 Biostatistics Resource, Keck Laboratory, Yale University, 300 George Street, New Haven, Connecticut, USA; 5 Department of Genetics, Yale University School of Medicine, 333 Cedar Street, P.O. Box 208005, New Haven, Connecticut 06520-8005, USA

## Abstract

The interaction among multiple genes and environmental factors can affect an individual's susceptibility to disease. Some genes may not show strong marginal associations when they affect disease risk through interactions with other genes. As a result, these genes may not be identified by single-marker methods that are widely used in genome-wide association studies. To explore this possibility in real data, we carried out a two-stage model selection procedure of joint single-nucleotide polymorphism (SNP) analysis to detect genes associated with rheumatoid arthritis (RA) using Genetic Analysis Workshop 16 genome-wide association study data. In the first stage, the genetic markers were screened through an exhaustive two-dimensional search, through which promising SNP and SNP pairs were identified. Then, LASSO was used to choose putative SNPs from the candidates identified in the first stage. We then use the RA data collected by the Wellcome Trust Case Control Consortium to validate the putative genetic factors. Balancing computational load and statistical power, this method detects joint effects that may fail to emerge from single-marker analysis. Based on our proposed approach, we not only replicated the identification of important RA risk genes, but also found novel genes and their epistatic effects on RA. To our knowledge, this is the first two-dimensional scan based analysis for a real genome-wide association study.

## Background

In the past several years, genome-wide association studies (GWAS) have achieved great successes in identifying hundreds of genetic variants that affect dozens of complex diseases. Most studies reported to date primarily employed a single-marker-based analysis strategy. As multiple genetic variations and environmental risk factors are expected to jointly affect a complex phenotype, it is natural to ask whether there is any benefit from conducting a joint marker analysis, e.g., a systematic study of all possible pairwise interactions, versus single-marker analysis [1]. Both simulations [2,3] and analytical studies [4] indicate that an exhaustive two-dimensional (2-D) scan may have higher statistical power under certain genetic models, e.g., when certain epistatic effects exist. It is important to find whether real GWAS data have such epistatic patterns favoring a 2-D scan. To answer this question, we conducted a 2-D scan for a GWAS data set from the North American Rheumatoid Arthritis Consortium (NARAC) supplied by Genetic Analysis Workshop 16 (GAW16). We studied the extra information offered by 2-D scan and identified epistatic effects. Furthermore, we propose a two-stage analysis strategy that incorporates single-marker analysis, 2-D scan, and a multiple marker analysis using LASSO to balance statistical power and computational feasibility in GWAS analysis.

## Methods

In the first stage of our proposed method for joint marker analysis, single-nucleotide polymorphisms (SNPs) are screened by using both a marginal search (single-marker analysis) and 2-D scan. For the marginal search, the simple logistic regression model is employed for each SNP *j *as follows:

The 2-D scan evaluates all possible SNP pairs by using the following additive models and interaction models:

where 1 ≤ *j *≤ *k *≤ *p *index SNPs, genotype values *X*_*j *_and *X*_*k *_= 0, 1, or 2 denote the number of the minor allele at each SNP. The overall statistical significance of Models (1), (2), and (3) measures the significance of the *marginal effect *of SNP *j*, the *additive joint effects *of SNPs *j *and *k*, and the *complete joint effects *of SNPs *j *and *k*, respectively. In Model (2), the statistical significance of parameters α_1*jk *_(or α_2*jk*_) measures the *conditional additive effects *of SNP *j *(or *k*), given SNP *k *(or *j*). In Model (3), the significance of  measures the *interaction effect *(epistasis) between SNPs *j *and *k*. The corresponding log-likelihood-ratio test statistics (LLR) quantify the statistical significance of models and parameters and thus the corresponding genetic effects. We wrote a C-program to implement logistic regression analysis, allowing for the exhaustive 2-D search (this program is available upon request from the authors). We chose the SNPs from the models that were ranked highest based on LLR.

After the first stage analysis identifies a set of candidate SNPs and SNP interactions, we apply the LASSO model selection method [5,6] to select predictive factors from those candidates.

In the LASSO model, the variables to be considered are either genotype values that reveal signals of marginal and conditional additive effects, or the products of genotype values, i.e., interaction terms, which reveal the signals of epistases. We use the R package *glmnet *[7] for the logistic regression model selection.

## Results

### Data cleaning

For GAW16 data quality control, we excluded those SNPs whose Hardy-Weinberg equilibrium *p*-values < 0.001 or minor allele frequencies < 0.01, and also excluded SNPs or individuals with missing rates >10%. Outliers were removed based on principal component analysis. Consequently, the final data include 500,884 SNPs and 2,002 individuals (862 cases and 1,140 controls). In the first-stage analysis the missing observations of the corresponding SNP(s) were eliminated at each model fitting. In the second stage, we imputed the missing SNP genotypes using software Beagle [8].

### First stage

#### Marginal association in Model (1)

**Figure 1:**
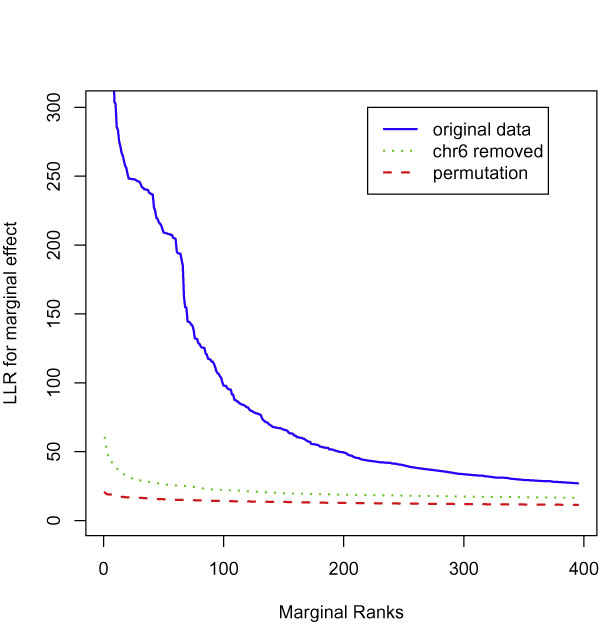
**Top log-likelihood test statistics for the marginal models**. Blue solid curve shows the 395 top values of marginal LLR from original data; the green dot curve shows the top marginal LLR excluding the SNPs in chr6; the red dash curve shows the top marginal 395 LLRs from all SNPs after permuting RA disease status.

There are 395 SNPs showing significant marginal effects with LLR > 27.04 (Bonferroni *p*-values < 0.1). Most of these SNPs are located in chromosome region 6p21, with high linkage disequilibrium (LD) existing among some of them. We sorted the test statistics of marginal association in decreasing order. The blue solid curve in Figure 1 exhibits the LLR values for these 395 SNPs. The green dot curve shows the top marginal LLRs without the SNPs in chromosome 6 (chr6), which contains the most signals for rheumatoid arthritis (RA) as reported in the literature. As a reference baseline, the red dash curve shows the top 395 values from the marginal LLRs of all SNPs when the disease status is permuted, i.e., when no association exists between RA and any SNP in the whole data set. Because the green dot curve is above the red dash curve, it suggests that additional marginal association signals exist outside of chr6.

#### Conditional additive effect in Model (2)

Because a two-marker model can be statistically significant if one of the SNPs has an extremely high marginal association, we did not study the conditional additive effects of the top 200 marginally associated SNPs (LLR > 49) in Model (2). Excluding these 200 SNPs, 71,693 two-marker full models in Form (3) (with 18,391 unique SNPs) were found to be significant with LLR > 59.37 (Bonferroni *p*-value < 0.1). Hierarchically nested within these full models, 70,795 two-marker additive models in Form (2) contain at least one SNP with a conditional additive effect that has an LLR > 27.04 (Bonferroni *p*-value < 0.1). These additive models involve 18,388 unique SNPs, 11.46% of which are on chr6.

**Figure 2:**
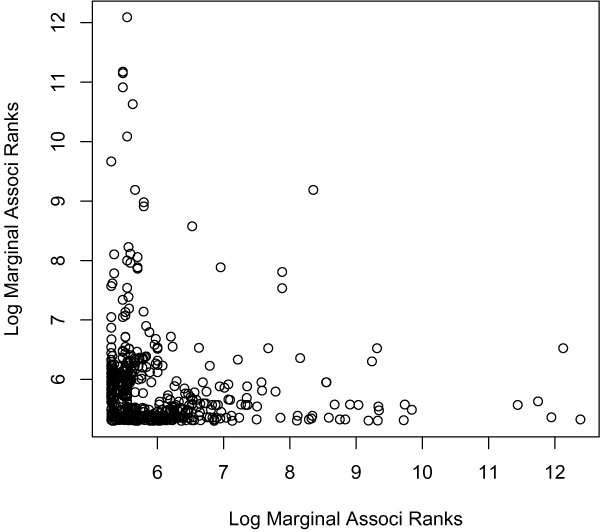
**Log marginal association ranks for SNP pairs with significant conditional additive effect**. Each dot represents a SNP pair (totally 494 SNP-pairs). Displacement of a dot along the x- and y-axes indicate the log of the marginal association ranks for the corresponding two SNPs. A lower marginal rank indicates a larger marginal effect.

A SNP can show a significant conditional additive effect given many other different SNPs. To avoid duplications, we included only one SNP pair that has the targeted SNP showing significant conditional additive effect. We obtained 494 pairs of SNPs that consisted of 506 unique SNPs (75.3% are from chr6, 505 have significant conditional additive effect). To illustrate the connection between conditional additive effects and marginal effects, Figure 2 shows the log-transformed marginal association ranks of the two SNPs in each pair. Almost all of the SNP-pairs include at least one SNP with relatively large marginal effect indicated by lower marginal ranks. This fact implies the existence of two possible situations: a SNP with large marginal effect may also exert a large conditional additive effect; or, a SNP with a small marginal effect can contribute a significant additive association given another SNP that has a large marginal effect. To check the prevalence of the second situation in our analysis, 33 of the 505 conditionally significant SNPs actually show small marginal effect (with LLRs for single-marker model <10, or the marginal ranks >4300).

**Figure 3:**
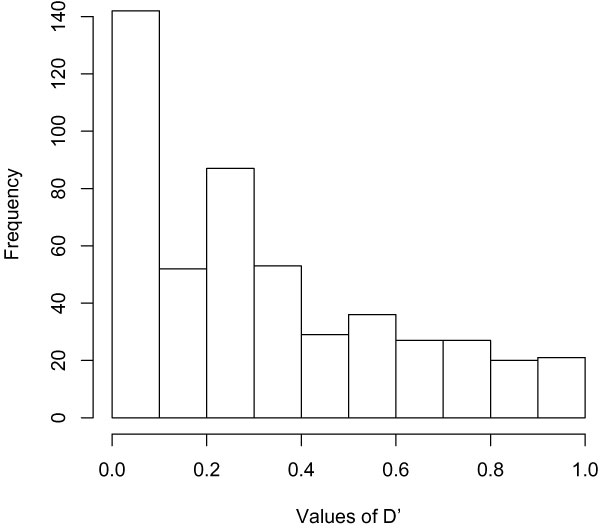
**Histograms of D' of SNP pairs with significant conditional additive effect**. Bars show the distribution of the number of SNP-pairs (totally 494 SNP pairs) over the values of D'. A large D' indicates significant linkage disequilibrium between two SNPs.

To illustrate the LD pattern of the SNP pairs that have large conditional additive effects, Figure 3 shows the histogram of D' of the chosen 494 SNP-pairs. Most of the SNP-pairs have relatively strong LD (60.73% have D' > 0.2, by the R package *genetics *[9]). In particular, for the above-mentioned 33 SNP pairs containing SNPs with small marginal but large conditional effects, their LDs are all significant (D' > 0.39). Because this LD pattern indicates that these SNP pairs are physically spaced closely, their significant conditional additive effects may represent haplotype effects.

#### Epistasis in Model (3)

**Figure 4:**
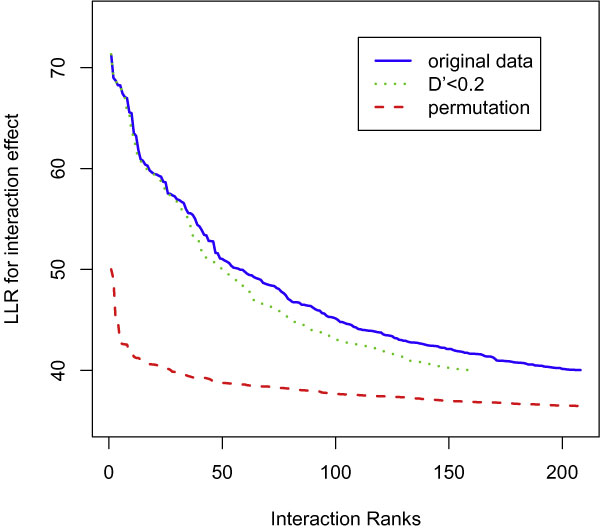
**Top log-likelihood test statistics for the large interaction effects**. The blue solid curve shows the top 208 decreasingly ordered LLR test statistics, among which 160 values corresponds to SNP-pairs that exist on different chromosomes or have D' < 0.2, as represented by the green dot curve. The red dash curve shows the top 208 LLR test statistics for the interaction terms after a permutation of the disease status.

**Figure 5:**
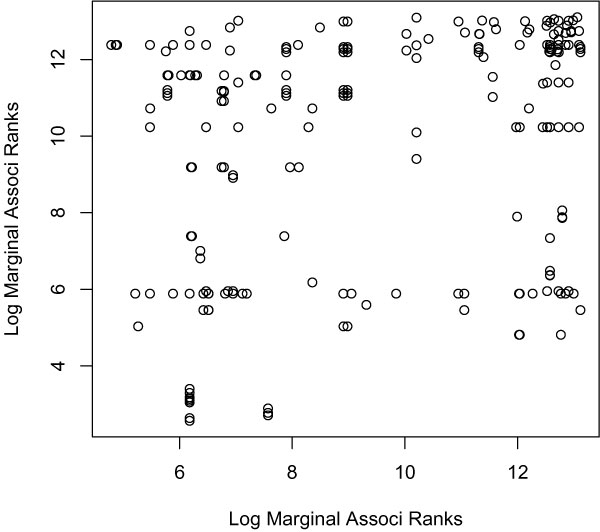
**Marginal association ranks of SNP pairs with large interaction effects**. Each dot represents a SNP pair (totally 208 SNP pairs). Displacement of a dot along the x- and y-axes indicate the log of the marginal association ranks for the corresponding two SNPs.

Epistasis is another type of joint effect, which is represented by the interaction term in Model (3). The top 208 epistatic terms have their corresponding LLRs > 40, or unadjusted *p*-values < 2.54 × 10^-10^. Of these 208 interactions, 46 are significant (LLR > 52.78; Bonferroni *p*-values < 0.047. In Figure 4, the blue solid curve shows the values of these 208 LLR in decreasing order. Among these 208 epistatic terms, 160 (or 196) terms (as illustrated in the green dot curve) involve the SNP pairs that either exist in different chromosomes or have D' < 0.2 (or 0.4). The red dash curve represents 208 top LLR values measuring the best interaction terms in the null scenario that is obtained from fitting all SNP pairs to a permuted RA status. The difference between the red dash curve and the blue solid curve indicates the presence of epistasis. Because the green dot curve has excluded the pairs of SNPs likely located in the regions of strong LD, the closeness between the blue solid and green dot curves suggests that most of these identified epistatic effects are not likely due to haplotype effects. This means that even though these interactive SNPs are mostly located within a chromosome (84.6% are in chr6), haplotype analysis has limited power to find these epistatic effects discovered through an exhaustive 2-D search. For these top 208 epistatic pairs, Figure 5 demonstrates the log-transformed ranks of their marginal effects, and shows that many SNPs have strong interactions but small marginal effects. Following this, we expect that genome-wise 2-D screening may be more informative than the marginal single-marker screening.

### Second stage

The goal of the second stage is to jointly select from the candidates: first, the top 395 SNPs with significant marginal associations in Model (1); second, the 506 unique SNPs from the 494 SNP pairs with significant conditional additive effects in model (2); and third, the top 208 epistatic SNP pairs by Model (3). In total, 914 variables were selected as input variables for LASSO selection, in which 706 variables were the genotypes of non-overlapping SNPs; 208 variables were the cross-products of genotype values of the 208 SNP-pairs.

To obtain a model that associates RA disease status with SNPs [7]. Bayesian information criterion (BIC) was used as the criterion to choose the tuning parameter λ. The LASSO model selection generated 63 non-zero coefficients, all for the SNPs from Models (1) and (2). LASSO did not pick up any interactions that represent epistasis. The result (available upon request) contains genes reported in the literature where marginal association studies were applied, i.e., *PTPN22 *(rs2476601), *HLA *genes, and *C5 *(rs2900180) [10-13]. Furthermore, it also includes many genes showing relatively small marginal association but significant joint effects when studied together with the other genes.

### Validation

#### Validate the two-stage method selection with WTCCC data

Using the Wellcome Trust Case Control Consortium (WTCCC) data [11], we sought to validate the genes indentified in the two-stage method with the GAW16 data. SNPs in the GAW16 data were mapped to genes through the SNP annotation file provided by Plenge et al. [12]. The genes were then associated with WTCCC SNPs based on the gene information downloaded from NCBI [14]. Fifty-seven genes were located by the 63 selected GAW16 SNPs showing large marginal or conditional additive effects. However, the two genes *LOC389362 *and *C14orf151 *(and their aliases) among those 57 genes are not represented in the WTCCC data. Within the rest of genes (or around ± 5 kbp if no SNP is found within the gene), we retrieved WTCCC SNPs. Again, missing genotypes were imputed by Beagle [8].

We got 1,371 WTCCC SNPs from the 61 genes. Their genotypes were fed as candidates into the LASSO model selection. The number of SNPs selected by LASSO depends on the value of tuning parameter λ. In order to guarantee that the LASSO-selected SNPs are statistically significant as a whole set, we chose the value of λ that led to the average number of false positive predictors to be less than 0.05 under the null hypothesis of no association. Specifically, with the selected value of λ, we permuted the responses for 1,000 times and obtained an average model size of 0.05. Table 1 summarizes the jointly selected genetic factors associated with RA by LASSO. Large marginal ranks of some identified SNPs indicate the single-marker analysis cannot find these SNPs at a reasonable significant level. Corresponding to these found SNPs, gene *PTPN22 *and the major histocompatability complex (MHC) region genes *HLA *and *BTNL2 *reported in the literature are also contained in our results. Gene *C6orf10 *is located in the MHC region, but to the best of our knowledge was not previously reported as RA risk genes. Associated genes *PGCP *and *MYO18B *are in novel regions on 8q22.2 and 22q11.1, respectively.

**Table 1:** WTCCC-data-validated SNPs and SNP pairs (epistases) associated with RA

Chr	SNP	Location	Gene Code	± kbp^a^	OR^b^	*p*-Value^c^	Marg. Rank^d^
1	rs3811019	114183625	*PTPN22*	0	1.46	6.93 × 10^-7^	1150
6	rs1265777	32381136	*C6orf10*	0	1.01	9.84 × 10^-1^	436
6	rs574710	32396168	*C6orf10*	0	0.89	7.44 × 10^-1^	488
6	rs539703	32396440	*C6orf10*	0	1.05	9.34 × 10^-1^	440
6	rs2894249	32433813	*C6orf10*	0	0.79	3.76 × 10^-4^	245
6	rs2076533	32471505	*BTNL2*	0	2.03	2.00 × 10^-16^	630
6	rs3763308	32482618	*BTNL2*	0	0.42	1.91 × 10^-8^	959
6	rs9268645	32516505	*HLA-DRA*	0	0.85	2.90 × 10^-2^	278
6	rs7194	32520458	*HLA-DRA*	0	1.02	7.96 × 10^-1^	110
6	rs9273363	32734250	*HLA-DQB1*	5	0.73	4.14 × 10^-9^	709
6	rs6908943	32743274	*HLA-DQB1*	5	0.69	2.92 × 10^-7^	1131
8	SNP_A-4193342	97922693	*PGCP*	0	1.38	1.68 × 10^-7^	416671
22	rs16981203	24729414	*MYO18B*	0	1.3	1.08 × 10^-5^	501

^a^± kbp, location of the SNPs. "0" indicates the SNP is physically located within the corresponding gene; "5" indicates the SNP is located outside the gene but is less than 5 kbp away.

^b^OR, the joint odds ratios and *p*-values in the full model containing all of the selected variables.

^c^*p*-value, for the full model containing all of the selected variables.

^d^Marg. Rank, marginal ranks of the SNPs by single-marker analysis in the WTCCC data. The rank >1015 corresponds to the Bonferroni *p*-value > 0.1 in a single-marker study.

#### Validation for pair-wise epistases with WTCCC data

We tried to validate the 103 gene-gene interactions (involving 91 unique genes) which were identified by the 208 most significant SNP pair epistases detected with Model (3) in the first stage of GAW16 data analysis. The WTCCC data were used to check whether significant epistases exist between SNPs from the corresponding gene pairs. Applying the same data quality control procedures as for GAW16 data, 1,781 unique SNPs were extracted from the WTCCC data and combined into 35,515 SNP-pairs according to the corresponding gene-pairs. Table 2 lists the validated significant gene-gene interactions (Bonferroni *p*-value < 0.05). These results show that the important gene-gene interactions for RA interactions are mostly located within the MHC region, but may reflect redundant information about the overlapped regions.

**Table 2:** SNP-pairs with large epistatic effects validated with WTCCC data

Chr1	SNP1	Location1	Gene1	Marg. Rank1^a^	Chr1	SNP1	Location1	Gene1	Marg. Rank2^a^	*p*-Value
6	rs2244579	31544618	*HCP5*	2933	6	rs206015	32290737	NOTCH4	623	1.39 × 10^-5^
6	rs4394275	31426156	*HLA-B*	4705	6	rs9276440	32822761	HLA-DQA2	3042	5.33 × 10^-5^
6	rs4394275	31426156	*HLA-B*	4705	6	rs9276432	32820362	HLA-DQA2	2165	1.06 × 10^-4^
6	rs4394275	31426156	*HLA-B*	4705	6	rs9276429	32820082	HLA-DQA2	2386	1.08 × 10^-4^
6	rs2248880	31341489	*HLA-C*	130792	6	rs9273363	32734250	HLA-DQB1	709	1.14 × 10^-4^
6	rs4394275	31426156	*HLA-B*	4705	6	rs9276431	32820225	HLA-DQA2	2484	1.40 × 10^-4^
6	rs9263794	31237998	*TCF19*	4325	6	rs438475	32294223	NOTCH4	1243	7.56 × 10^-4^
6	rs1265074	31221193	*CCHCR1*	20388	6	rs438475	32294223	NOTCH4	1243	2.20 × 10^-3^
6	rs2244579	31544618	*HCP5*	2933	6	rs438475	32294223	NOTCH4	1243	2.27 × 10^-3^
6	rs4394275	31426156	*HLA-B*	4705	6	rs9273363	32734250	HLA-DQB1	709	3.49 × 10^-3^
6	rs2844615	31350938	*HLA-C*	10590	6	rs2596477	31435702	HLA-B	76640	2.36 × 10^-2^
6	rs4394275	31426156	*HLA-B*	4705	6	rs2227127	32819760	HLA-DQA2	585	3.16 × 10^-2^
6	rs2736172	31698877	*BAT2*	406	6	rs438475	32294223	NOTCH4	1243	3.37 × 10^-2^
6	rs1063635	31487910	*MICA*	897	6	rs206015	32290737	NOTCH4	623	3.41 × 10^-2^

Each row gives the annotations of SNP-pairs with validated epistatic effects.

^a^Marg. Rank 1 and 2 give the ranks of single-marker association strengths of the SNPs 1 and 2 in the WTCCC data. The rank > 1015 corresponds to the Bonferroni *p*-value > 0.1 in a single-marker study.

## Discussion

Joint SNP analysis can benefit GWAS more than single-SNP analysis in at least two aspects. First, in GWAS many strong marginal associations are likely due to strong LD with a truly associated locus. So single-marker analysis may pick up many SNPs but mostly they are nested within one or two narrow genomic regions. In joint analysis, e.g., LASSO selection, SNPs that have high correlation with those already included in the model are less likely to be added into the model again. This may help us to study more interesting regions while keeping in mind the hotspots. In other words, if we retain the same number of SNPs for follow-up studies, joint analysis likely brings a wider genome region into further consideration. Second, joint analysis can identify truly associated predictors that have small marginal signals but large conditional additive effects or large epistatic effects. Empirical data suggest this scenario does exist. These findings are potentially valuable in further exploring the relationships among genes in pathway studies.

Two important issues should be noted with regard to our methodology. First, LASSO tends to over-fit when choosing λ based on the BIC criterion. To illustrate this issue does exist, we permuted the disease status of the WTCCC data set in the validation stage, in which the BIC-controlled LASSO led to false-positive selection. To overcome this problem, permutation was used to determine an appropriate value for λ when quantifying the proportion of false positives. Second, in the second stage of selection, it may cause over-fit when isolated applying the significance control to the SNPs identified by screening the same data set. To prove this problem exists, we permuted the RA status before going through the whole two-stage analysis procedure for the GAW16 data set. In this case, even though no SNP is associated with the disease, LASSO still selected some variables, even if a λ value was chosen in the second stage in the same way as we did for WTCCC validation. To address this problem, we may similarly apply the same procedure using permutations at the outset of screening analysis to select an appropriate λ value for the original data. However, this requires intensive computation and may lead to fewer SNPs to be followed up in other studies. In practice, these issues can be alleviated by using a separate data set to validate the results. In this way we can carry out a screening and then apply LASSO, while properly controlling λ for the final model in the validation stage.

We reported our results using a Bonferroni *p*-values at the 0.1 level in the first stage because we would like to avoid missing true associations, and hope that the second stage analysis will be able to select terms according to a more stringent criterion. We have also tried the significance control level of 0.05 that led to slightly fewer gene findings (except obtaining one extra gene *C14orf151*) from the GAW16 data analysis. However, both control levels led to exactly the same set of detected signals shown in Table 1, after the validation procedure with the WTCCC data. Therefore, our method seems to be robust to the choice of the threshold levels in this range.

## Conclusion

In GWAS, there exist SNPs with small marginal but large joint associations with RA. To extract more information from GWAS data, we have proposed a two-stage association detection method based on an exhaustive two-dimensional screening and the LASSO model selection. Our method studies joint associations including gene-gene interactions. Applying this joint analysis method to GAW16 data and validating the results with a separate data set (WTCCC data), we have found novel genes associated with RA, as well as interactions implying complex RA associations in the MHC region.

## List of abbreviations used

2-D: Two-dimensional; BIC: Bayesian information criterion; Chr6: Chromosome 6; GAW16: Genetic Analysis Workshop 16; GWAS: Genome-wide association study; LD: Linkage disequilibrium; LLR: Log-likelihood-ratio; MHC: Major histocompatability complex; NARAC: North American Rheumatoid Arthritis Consortium; RA: Rheumatoid arthritis; SNP: Single-nucleotide polymorphism; WTCCC: Wellcome Trust Case Control Consortium

## Competing interests

The authors declare that they have no competing interests.

## Authors' contributions

ZW and HZ conceived and designed the analysis and wrote the manuscript. ZW contributed analysis tools and analyzed the data. CA, DHB, JYL, and JSL participated in the data preparation and analysis and helped to draft the manuscript. All authors read and approved the final manuscript.
